# Elimination of Proliferating Cells Unmasks the Shift from Senescence to Quiescence Caused by Rapamycin

**DOI:** 10.1371/journal.pone.0026126

**Published:** 2011-10-11

**Authors:** Olga V. Leontieva, Zoya N. Demidenko, Andrei V. Gudkov, Mikhail V. Blagosklonny

**Affiliations:** Department of Cell Stress Biology, Roswell Park Cancer Institute, Buffalo, New York, United States of America; Penn State Hershey Cancer Institute, United States of America

## Abstract

**Background:**

Depending on cellular context, p53-inducing agents (such as nutlin-3a) cause different outcomes including reversible quiescence and irreversible senescence. Inhibition of mTOR shifts the balance from senescence to quiescence. In cell lines with incomplete responses to p53, this shift may be difficult to document because of a high proportion of proliferating cells contaminating arrested (quiescent and senescent) cells. This problem also complicates the study of senescence caused by minimal levels of p21 that are capable to arrest a few cells.

**Methodology:**

During induction of senescence by low levels of endogenous p53 and ectopic p21, cells were co-treated with nocodazole, which eliminated proliferating cells. As a result, only senescent and quiescent cells remained.

**Results and Discussion:**

This approach revealed that rapamycin efficiently converted nutlin-induced-senescence into quiescence. In the presence of rapamycin, nutlin-*arrested* MCF-7 cells retained the proliferative potential and small/lean morphology. Using this approach, we also unmasked senescence in cells arrested by low levels of ectopic p21, capable to arrest only a small proportion of HT1080-p21-9 cells. When p21 did cause arrest, mTOR caused senescent phenotype. Rapamycin and high concentrations of nutlin-3a, which inhibit the mTOR pathway in these particular cells, suppressed senescence, ensuring quiescence instead. Thus, p21 causes senescence passively, just by causing arrest, while still active mTOR drives senescent phenotype.

## Introduction

Non-dividing (arrested) cells can be either quiescent or senescent [1], [2]. The senescent phenotype, driven in part by growth-promoting pathways such as mTOR [3], [4] is characterized by large/flat cell morphology (hypertrophy), a pro-inflammatory and hyper-secretory phenotype, beta-Gal-staining and permanent loss of proliferative potential [5]–[8]. Proliferative potential (PP) is not proliferation but a potential to proliferate [9]. Like senescent cells, quiescent cells do not proliferate but, unlike senescent cells, they retain PP. For example, cells can be arrested by nutlin-3a, which reversibly induces p53. When nutlin-3a is removed, quiescent cells re-start proficient proliferation, whereas senescent cells do not [10].

p53 can both cause and suppress the senescent phenotype. By causing cell cycle arrest, while not inhibiting cellular mass growth, p53 can cause senescence [11]. When p53 inhibits mTOR, it converts senescence into quiescence [11]. In several cancer cell lines, nutlin-3a failed to inhibit mTOR and some cells acquired senescent morphology [12]. However, senescent cells co-existed with proliferating cells that were not arrested by nutlin-3a. These proliferating cells rapidly overwhelmed the culture. To investigate proliferative potential (PP) of arrested cells, it seems prudent to eliminate all proliferating (nutlin-nonresponsive) cells. A similar problem complicates the study of p21-induced senescence. To observe whether cells retain the proliferative potential, one needs to induce p21 and then to switch it off. In a useful model (HT1080-p21-9 cells), HT-1080 cells express ectopic p21, inducible by IPTG. IPTG-induced p21 rapidly arrests cells, which become senescent in 3-4 days. By increasing the concentration of IPTG, one can increase levels of p21 to achieve senescence in all cells. However, at such high levels, p21 may exert other effects besides cell cycle arrest. It was suggested that p21 actively induces the senescent program, independently from its ability to cause arrest. Alternatively, p21 causes senescence passively, just by causing arrest (while still active growth-promoting pathways (such as mTOR) drive senescent phenotype). To distinguish between the two models, we wish to determine whether minimal levels of p21 (capable to arrest a small proportion of cells) still cause senescence in arrested cells. For that, we need to eliminate cells that are not arrested by p21.

Our unrelated studies suggested a simple solution, given that cell cycle arrest protects normal cells from chemotherapy with mitotic inhibitors [13]. Mitotic inhibitors such as nocodazole killed only proliferating cells, whereas both senescent and quiescent HT1080-p21-9 cells were spared [14].

## Results

### Treatment of nutlin-arrested cells with nocodazole

**Figure 1:**
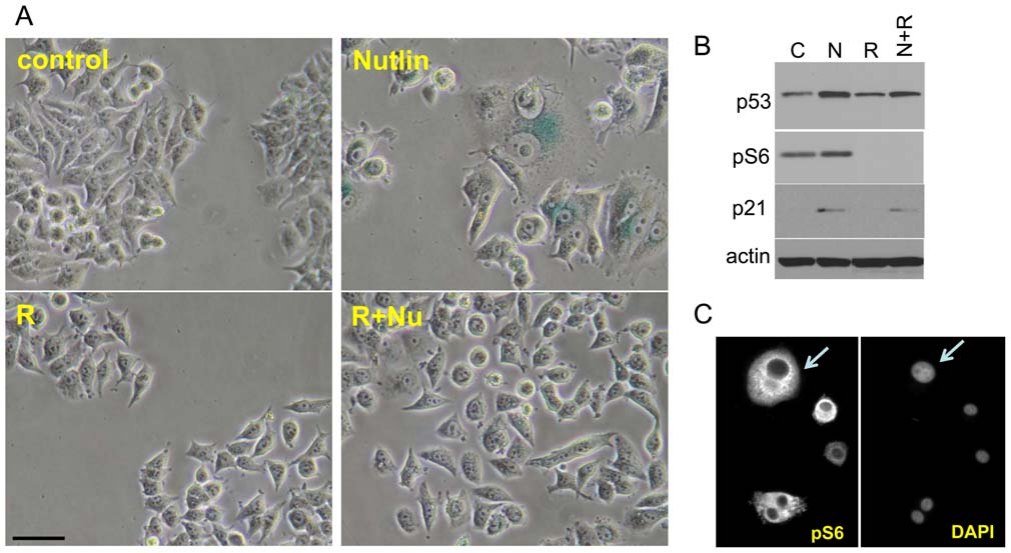
Rapamycin prevents nutlin-induced senescent morphology in MCF-7 cells. A. MCF7 cells were plated at 5000 or 10000/well in 12 well plates, allowed to attach and then were either pretreated with 500 nM rapamycin (R), or left untreated in complete medium (control). The next day, 5 µM nutlin-3a was added. After 5 days, cells were stained for beta-Gal and microphotographed (bar -50 micron). B. MCF7 cells were treated as indicated for 24 hr, and immunoblot was performed as described in Methods; C- control, N- 5 µM Nutlin 3a, R- 500 nM rapamycin. C. MCF7 cells were seeded in on-slide chambers at 500 cells/well with 5 µM Nutlin-3a After 4 days cells were fixed and stained for pS6; nuclei were visualized by counterstaining with Hoechst 33258 (DAPI). Photos were taken at 20x.

In MCF-7 cells, in agreement with our previous report [12], nutlin-3a caused senescent morphology in some but not all cells (Fig. 1A) and did not decrease levels of pS6, a marker of mTOR activity (Fig. 1B). Given that only a small proportion of cells become senescent, we also measured mTOR activity in individual cells by immunostaining (Fig. 1C). Most nutlin-3a-treated cells were positive for pS6; including all cells with a large, flat (senescent) morphology (Fig. 1C). Rapamycin blocked pS6 (Fig. 1B) and prevented senescent morphology in nutlin-treated cells (Fig. 1A). However, it was difficult to test proliferative potential (PP) of nutlin-arrested cells because still proliferating cells overwhelmed the cell culture. This masks potentially irreversible arrest in arrested cells.

**Figure 2:**
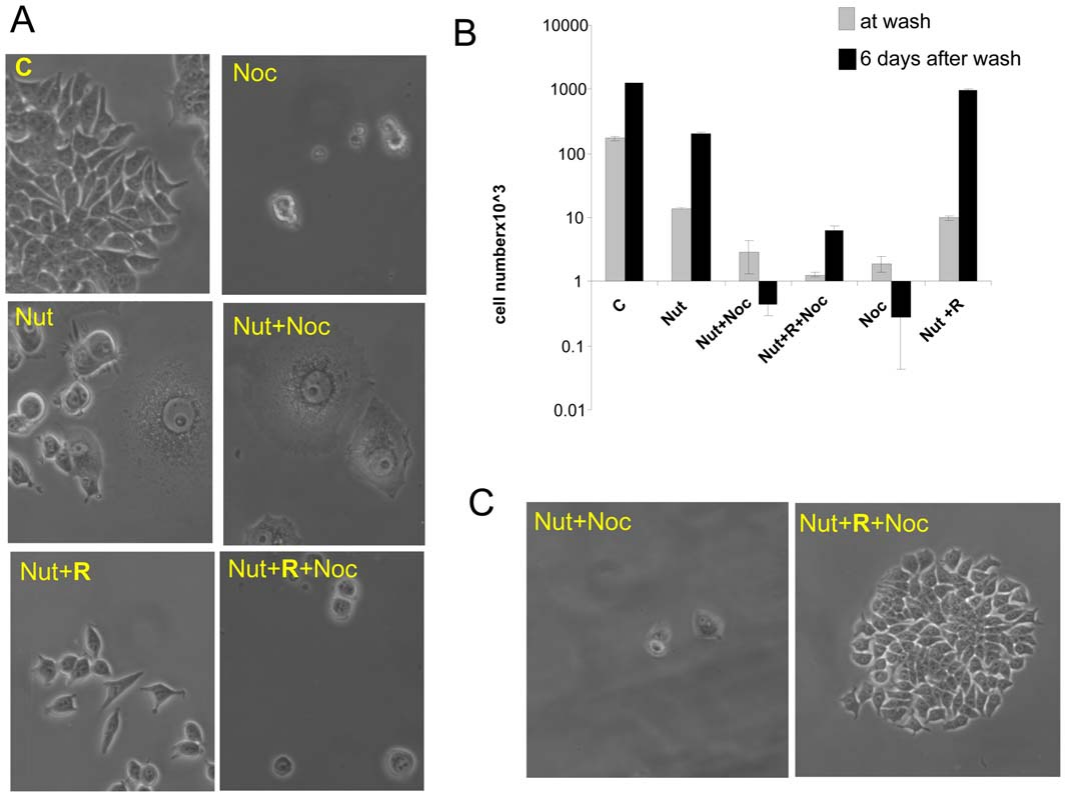
Co-treatment with nocodazole eliminates proliferating cells. A. MCF-7 cells were plated in 12-well plates and treated with 5 µM nutlin-3a (Nut) or 5 µM nutlin-3a + 10 nM rapamycin (Nut+R). The next day, 200 nM nocodazole was added as indicated (Noc) in the right panel. After 4 days live cells were microphotographed. B. MCF-7 cells were treated as described in panel A. After 4 days with nocodazole 1 set of cells was counted (at wash) and 2^nd^ set was washed and incubated for 6 days in drug free-medium and then live cells were microphotographed (C) and counted (after 6 days). Results are shown as cell numbers per well. C. Microphotographs of MCF-7 cells treated as described in B.

To eliminate cells that still proliferate in the presence of nutlin-3a, we co-treated the culture with nocodazole (Fig. 2). While eliminating proliferating cells (Noc), nocodazole spared quiescent (Fig. 2: Nu+Noc+R) and senescent (Fig. 2: Nut+Noc) cells (Fig. 2A). Rapamycin prevented senescent morphology in nutlin-arrested cells (Fig. 2A). These cells retained PP and resumed proliferation (Fig. 2B). In other words, rapamycin shifted senescence to quiescence. Like senescent cells, quiescent cells did not proliferate and therefore were spared from nocodazole. Unlike senescent cells, however, quiescent cells retained PP and formed colonies after all drugs were washed out (Fig. 2C).

### Treatment of IPTG-treated cells with nocodazole

**Figure 3:**
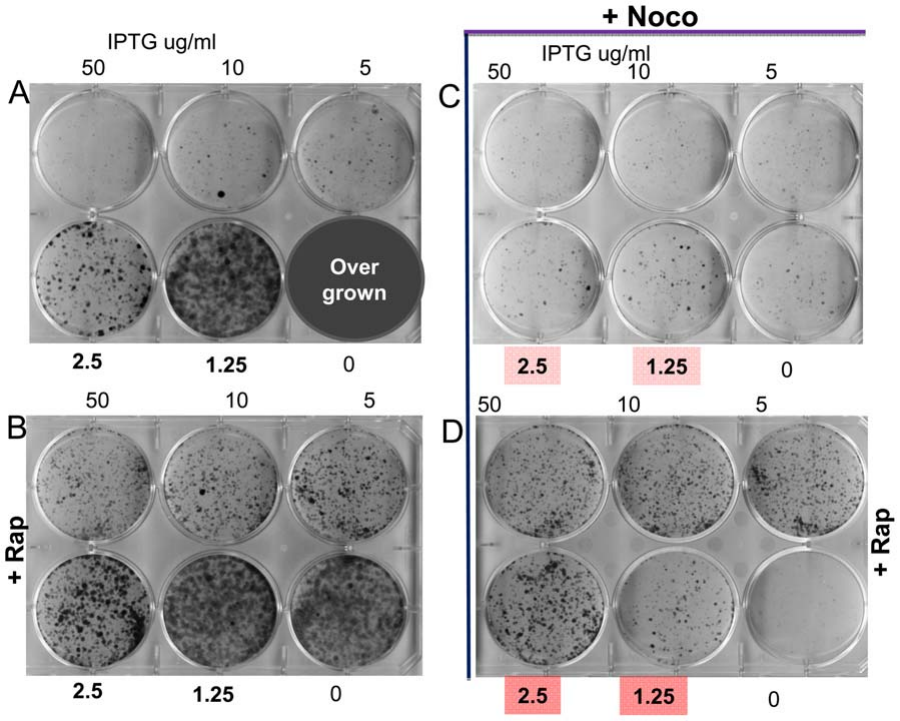
Rapamycin suppresses senescence caused by low concentrations of IPTG. HT1080-p21-9 cells were plated in 6-well plates at 5000 per well with a range of IPTG concentrations in the absence (A and C) or in the presence of 500 nM rapamycin (+Rap) (B and D). Next day 200 nM Nocodazole was added to half of the wells (right panel - +Noco). After 3 days, cells were washed and incubated in drug-free medium for 7 days. Then colonies were stained with crystal violet. Note: In the absence of any drugs, cells overgrew and could not be stained (overgrown).

**Figure 4:**
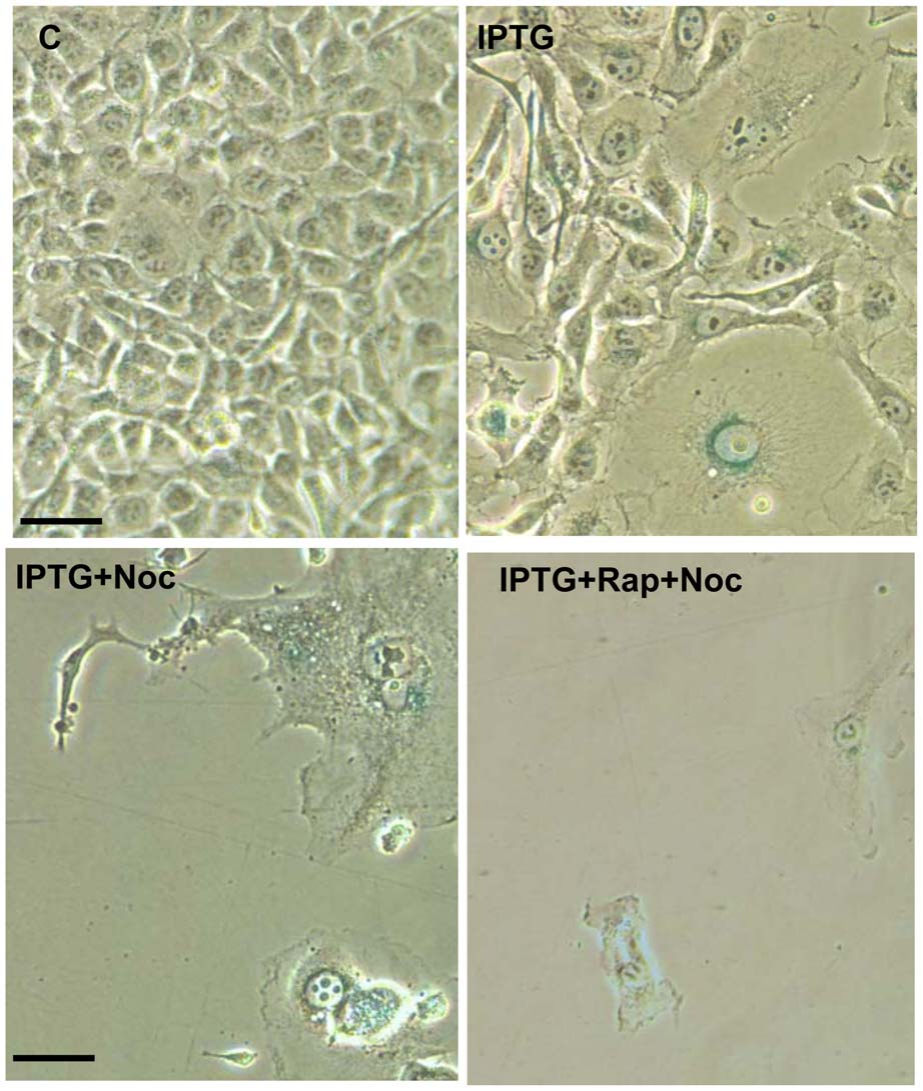
Co-treatment with nocodazole reveals the gerosuppressive effect of rapamycin at low concentrations of IPTG. HT1080-p21-9 cells were plated in 12 well plates at 20,000 cells per well and incubated with 1.25 µg/ml IPTG with or without 200 nM nocodazole (Noc) and in the presence or absence of 500 nM rapamycin (Rap) as indicated. After 3 days, cells were stained for beta-Gal and microphotographed. Bar - 50 micron.

In a dose-dependent manner (with a maximal effect at 10 µg/ml or 50 µM), IPTG induces p21 and causes cell cycle arrest in HT1080-p21-9 cells [15], [16]. When 10 µg/ml IPTG was washed out after 3 days, cells could not resume proliferation because they died in mitosis [16]. As shown here, only about 5% of cells treated with 5–50 µg/ml IPTG formed colonies upon removal of IPTG (Fig. 3A). The number of colonies was progressively increased, when concentration of IPTG was decreased to 1.25 µg/ml, probably either because the arrest was reversible or because cells simply were not arrested by low concentrations of IPTG in the first place. In the presence of 1.25 µg/ml IPTG, some cells acquired senescent morphology (Fig. 4). But do these cells retain proliferative potential? Treatment with rapamycin did not allow us to answer this question clearly. Whereas at high concentrations of IPTG, rapamycin increased a number of colonies 10 fold (prevention of senescence), there was no detectable change in number of colonies at 1.25 µg/ml IPTG (Fig. 3B). At low concentrations of IPTG, the effect of rapamycin became less evident (Fig. 3B). Either low concentrations of IPTG caused quiescence rather than senescence or simply the culture was overwhelmed by proliferating cells, which did not respond to IPTG.

By eliminating non-arrested cells, nocodazole revealed the status of arrested cells (Fig. 3 C-D). At 0-2.5 µg/ml IPTG without rapamycin, only cells with senescent morphology survived nocodazole (Fig. 3C) and only a few colonies were formed. Rapamycin prevented the senescent morphology (Fig. 4) and also increased a number of colonies at all concentrations of IPTG, proportionally to a number of IPTG-arrested cells (Fig 3D). We extended treatment with nocodazole to 4 days (Fig. S1). At all concentrations of IPTG, including 1.25 µg/ml, cells poorly proliferated after removal of drugs (IPTG+Noc). Rapamycin increased cell numbers at all concentrations of IPTG (Fig. S1). We conclude that IPTG-arrested cells were senescent at all concentrations of IPTG.

**Figure 5:**
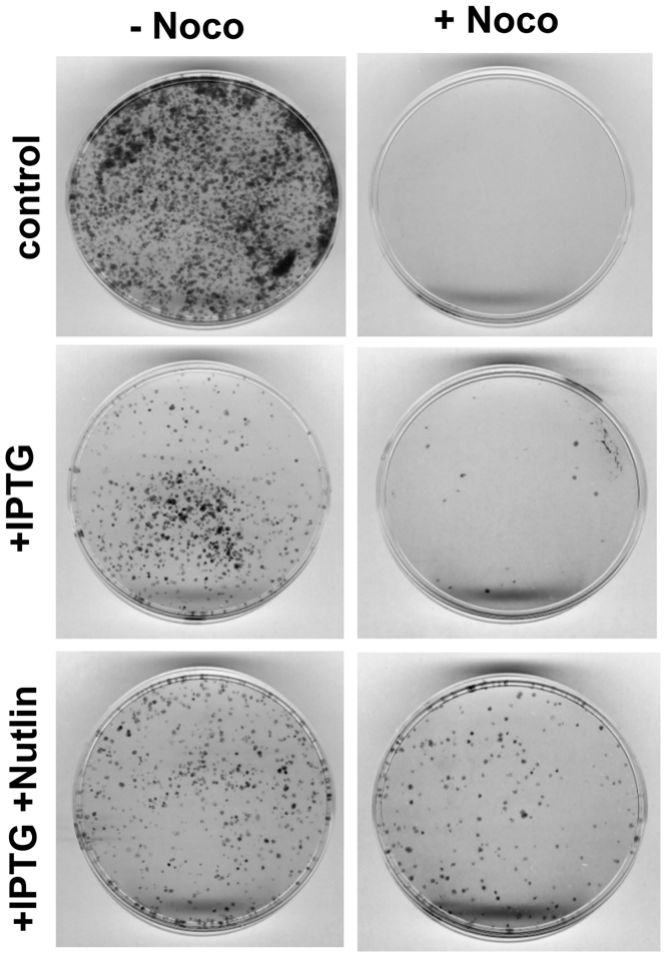
Nutlin-3a suppresses senescence caused by low concentrations of IPTG. HT1080-p21-9 cells were plated in 60 mm dishes at 5000 per dish with 2 ug/ml IPTG with or without 10 uM nutlin-3a as indicated. After 1 day, dishes on the right were treated with 200 nM nocodazole. After 3 days, cells were washed and incubated in drug-free medium for 4 days in control (to avoid overgrowth) and for 7 days for all other conditions. Then colonies were stained with crystal violet.

We have previously shown that nutlin-3a suppressed senescence caused by high concentrations of IPTG [10], [11]. Here we investigated whether nutlin-3a would also suppress senescence caused by low concentrations of IPTG. Cells arrested (and thus protected from nocodazole) by 2 µg/ml IPTG did not retain proliferative potential and did not form colonies, when these drugs were removed (Fig. 5). In such IPTG-arrested cells, nutlin-3a preserved proliferative potential (Fig. 5).

## Discussion

Here we extended the model that cellular senescence is caused by active nutrient-sensing and growth-promoting pathways such as mTOR, when the cell cycle is blocked by either p21 or p53. Regardless of whether arrest is induced by p21 or p53, it is the status of the mTOR pathway that in part determined senescence. In our previous work, it was crucial to arrest all cells. There were 2 limitations. First, in some cell lines, the arrest could not be achieved in all cells, unless toxic concentrations of p53-inducing agents were used. Second, in order to arrest all cells, ectopic p21 was used at super-physiological levels. Here we used low levels of p53 and p21 and achieved arrest in all cells, simply by eliminating non-arrested cells with nocodazole.

First, we found that at low concentrations of IPTG, while most cells were proliferating, the arrested cells were senescent. This supports the notion that the “senescent state” borders with the “growth state” [17]. Second, rapamycin shifted senescence to quiescence. Third, rapamycin did not force proliferation in the presence of IPTG but simply preserved PP. Fourth, senescent morphology (such as hypertrophy and beta-Gal staining, enlarged nucleoli) correlated with loss of PP. Fifth, this provides a model to study a pure senescent cell population. In brief, this work suggests that the nature of cell cycle arrest (whether it is caused by high or low levels of p21 and p53) does not determine senescent phenotype. Rather it is active growth-promoting mTOR-centered pathways that drive senescence in arrested cells.

## Materials and Methods

### Cell lines and reagents

HT1080-p21-9 cells, derived from HT1080 human fibrosarcoma cells (ATCC, Manassas, VA), were kindly provided by Dr. Igor Roninson and previously described [4], [7], [15], [16]. In these cells p21 expression can be turned on or off using a physiologically neutral agent isopropyl-thio-galactosidase (IPTG). MCF-7 breast carcinoma cell line was obtained from ATCC (Manassas, VA). MCF-7, breast cancer cell line was cultured in high-glucose DMEM (plus pyruvate) with 10% FBS. HT1080-p21-9 cells were cultured in high-glucose DMEM without puruvate supplemented with FC2 serum (HyClone FetalClone II from Thermo Scientific, Logan, Utah). In HT1080-p21-9 cells, p21 expression can be turned on or off using isopropyl-thio-galactosidase (IPTG) [16]. Rapamycin was obtained from LC Laboratories (MA, USA) and dissolved in DMSO as 5 mM solution. Nutlin-3a and nocodazole were purchased from Sigma-Aldrich (St. Louis, MO) and dissolved in DMSO as 10 µM and 6 mM stocks, respectively. IPTG (Invitrogen) was dissolved in water as 50 mg/ml stock solution and used in cell culture at a final concentration of 1.25-50 µg/ml.

### Immunoblot analysis

Whole cell lysates were prepared using boiling lysis buffer (1%SDS, 10 mM Tris.HCl, pH 74.). Equal amounts of proteins were separated on 10% or gradient polyacrylamide gels and transferred to nitrocellulose membranes. The following antibodies were used: mouse anti-p53 (Ab-6) from Oncogene, mouse anti-p21 from BD Biosciences; rabbit anti-actin from Sigma-Aldrich; rabbit anti-phospho-S6 (Ser235/236) from Cell Signaling Biotechnology. Secondary anti-rabbit and anti-mouse HRP conjugated antibodies were from Cell Signaling Biotechnology. Signals were visualized using ECL chemilumenescence kit from Pierce.

### SA-β-Gal staining

Beta-Gal staining was performed using Senescence-galactosidase staining kit (Cell Signaling Technology) according to manufacturer's protocol. Cells were incubated at 37°C until beta-gal staining becomes visible. Development of color was detected under light microscope [12].

### Proliferative potential

Proliferative potential was determined as described in detail in Figure legends.

### Immunostaining

Cells were fixed in 4% paraformaldehyde for 15 min, washed in PBS and permeabilized in 0.5% Triton X-100/PBS for 30 min at RT, blocked with 3% BSA/PBS for 30 min at RT followed by consequent incubations with 1∶200 dilution of rabbit anti-phospho S6 (Ser 235/236) antibody in 3% BSA/PBS for 1 h and secondary donkey anti-rabbit Alexaflour 594 antibody (Invitrogen) (1∶500) for 30 min. After washes cells were counterstained with Hoechst 33258 1 µg/ml in PBS for 15 min and mounted with antifade reagent Fluoromount G (Invitrogen).

### Colony formation assay

Cells in wells were fixed and stained with 1.0% crystal violet in 10% ethanol.

## Supporting Information

Figure S1 **Co-treatment with nocodazole reveals the gerosuppressive effect of rapamycin at low concentrations of IPTG.** HT1080-p21-9 cells were plated in 48-well plates and treated with indicated concentrations of IPTG in the absence (blue bars) or presence of rapamycin (red bars). Then 200 nM nocodazole was added and cells were cultured for 4 days. Cells were washed and incubated in drug-free medium for 7 days and the number of viable cells were determined by Celltiter blue reagent (Promega).
(TIF)
