# Supplementary figures and images for: Elimination of Proliferating Cells Unmasks the Shift from Senescence to Quiescence Caused by Rapamycin
Source: PLoS One. 2011 Oct 11;6(10):e26126. doi: 10.1371/journal.pone.0026126 (PMC3191182; doi:10.1371/journal.pone.0026126)

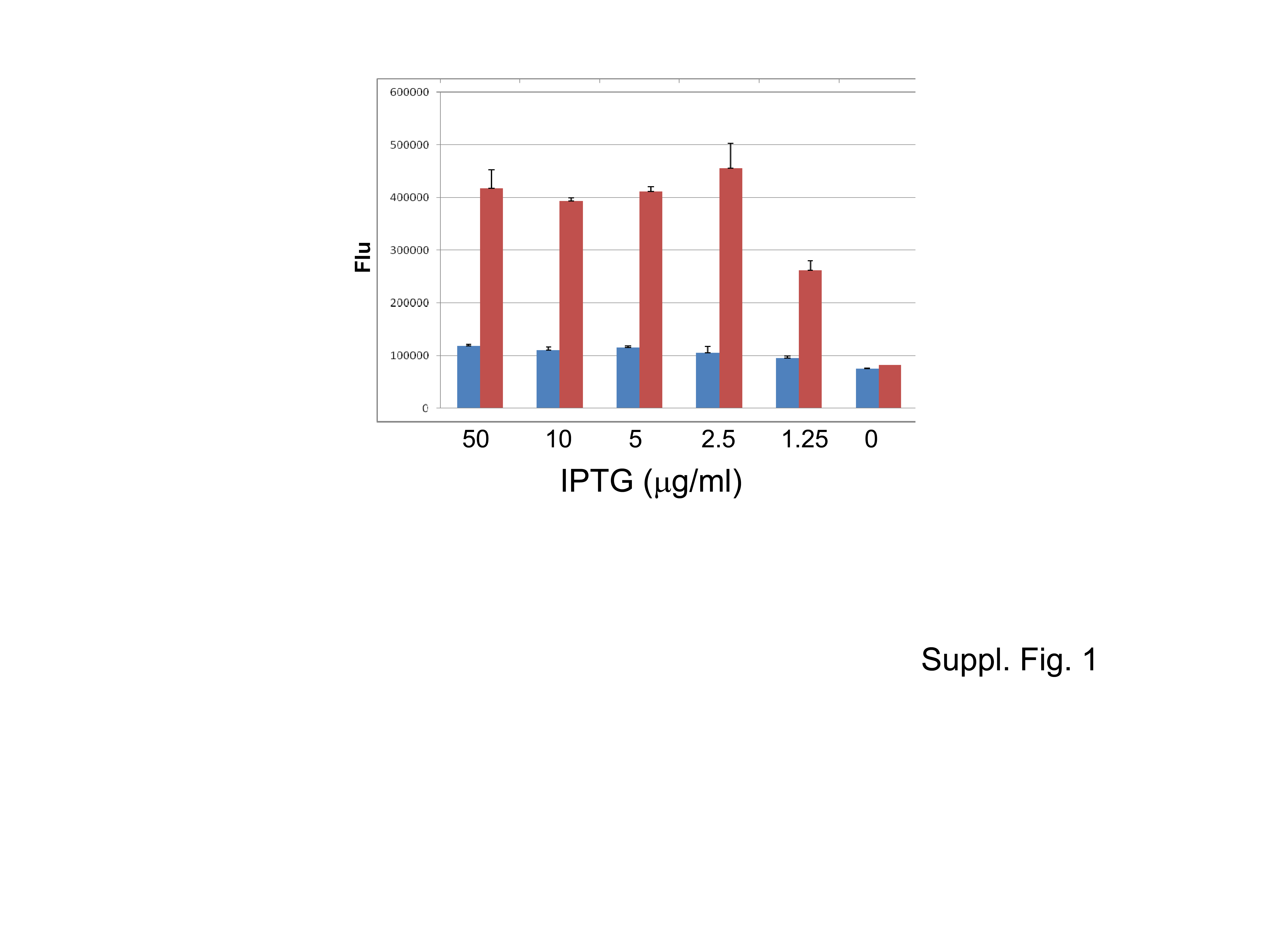

Supplement: Figure S1 — Co-treatment with nocodazole reveals the gerosuppressive effect of rapamycin at low concentrations of IPTG. HT1080-p21-9 cells were plated in 48-well plates and treated with indicated concentrations of IPTG in the absence (blue bars) or presence of rapamycin (red bars). Then 200 nM nocodazole was added and cells were cultured for 4 days. Cells were washed and incubated in drug-free medium for 7 days and the number of viable cells were determined by Celltiter blue reagent (Promega). (TIF) [file pone.0026126.s001.tif]
